# APTIMA assay on SurePath liquid-based cervical samples compared to endocervical swab samples facilitated by a real time database

**DOI:** 10.4103/1742-6413.65057

**Published:** 2010-07-02

**Authors:** Samer N. Khader, Kathie Schlesinger, Josh Grossman, Richard I. Henry, Mark Suhrland, Amy S. Fox

**Affiliations:** Address: Division of Cytopathology, Department of Pathology, Montefiore Medical Center, The University Hospital for the Albert Einstein College of Medicine, Bronx, NY, USA; 1 Division of Virology, Department of Pathology, Montefiore Medical Center, The University Hospital for the Albert Einstein College of Medicine, Bronx, NY, USA

**Keywords:** APTIMA combo 2, Chlamydia trachomatis, liquid based cytology, Neisseria gonorrhoeae, SurePath

## Abstract

**Background:**

Liquid-based cytology (LBC) cervical samples are increasingly being used to test for pathogens, including: HPV, *Chlamydia trachomatis* (CT) and *Neisseria gonorrhoeae* (GC) using nucleic acid amplification tests. Several reports have shown the accuracy of such testing on ThinPrep (TP) LBC samples. Fewer studies have evaluated SurePath (SP) LBC samples, which utilize a different specimen preservative. This study was undertaken to assess the performance of the Aptima Combo 2 Assay (AC2) for CT and GC on SP versus endocervical swab samples in our laboratory.

**Materials and Methods:**

The live pathology database of Montefiore Medical Center was searched for patients with AC2 endocervical swab specimens and SP Paps taken the same day. SP samples from CT-and/or GC-positive endocervical swab patients and randomly selected negative patients were studied. In each case, 1.5 ml of the residual SP vial sample, which was in SP preservative and stored at room temperature, was transferred within seven days of collection to APTIMA specimen transfer tubes without any sample or patient identifiers. Blind testing with the AC2 assay was performed on the Tigris DTS System (Gen-probe, San Diego, CA). Finalized SP results were compared with the previously reported endocervical swab results for the entire group and separately for patients 25 years and younger and patients over 25 years.

**Results:**

SP specimens from 300 patients were tested. This included 181 swab CT-positive, 12 swab GC-positive, 7 CT and GC positive and 100 randomly selected swab CT and GC negative patients. Using the endocervical swab results as the patient’s infection status, AC2 assay of the SP samples showed: CT sensitivity 89.3%, CT specificity 100.0%; GC sensitivity and specificity 100.0%. CT sensitivity for patients 25 years or younger was 93.1%, versus 80.7% for patients over 25 years, a statistically significant difference (*P* = 0.02).

**Conclusions:**

Our results show that AC2 assay of 1.5 ml SP samples transferred to APTIMA specimen transfer medium within seven days is sufficiently sensitive and specific to be used to screen for CT and GC. CT sensitivity may be somewhat reduced in samples from patients over 25 years. SP specimens retained in the original SP fixative for longer time intervals also may have decreased sensitivity, due to deterioration of RNA, but this was not assessed in this study. The ability to tap the live pathology database is a valuable tool that can useful to conduct clinical studies without a costly prospective clinical trial.

## INTRODUCTION

*Chlamydia trachomatis* (CT) is one of the most common sexually transmitted diseases, affecting an estimated 3 million sexually active adolescents and young adults annually in the United States.[1] The major problem with CT, as with *Neisseria gonorrhoeae* (GC), is that most women with a lower genital tract infection have no symptoms. Hence, the disease goes undetected, increasing the risk of spread to others through sexual contact and spread to the upper genital tract and possible sequelae of pelvic inflammatory disease, ectopic pregnancy, and infertility. Because of these serious complications of a disease that is often asymptomatic, annual screening for CT infection is now recommended for all sexually active women up to 25 years of age and for women older than 25 years who are high risk.[1]

This routine annual screening for CT is feasible because of the development of nonculture tests for chlamydia that are easy to perform, economical, and accurate. Of the tests available, Gen-Probe’s APTIMA AC2 nucleic amplification assay for CT and GC (Gen-Probe Inc., San Diego, CA), which is based on detecting rRNA, is among the most sensitive and specific.[2] At the same time, the increased use of liquid-based cytology (LBC) to screen for cervical cancer has opened the door to performing multiple tests, including CT and GC nucleic amplification tests, on a single cervicovaginal sample.[3–10] This is easier on the patient and convenient for the clinician. Most studies that have evaluated the accuracy of such testing on LBC have evaluated the ThinPrep Pap (Cytyc, Boxborough, MA), which is currently approved by the Food and Drug Administration (FDA) for AC2 testing. One large prospective multicenter study and a subsequent smaller study have evaluated the detection of CT and GC by AC2 on SP samples.[9,10] To validate the performance of AC2 testing on SP Paps at our institution we performed a similar comparison. This study, which was IRB approved, was facilitated by a daily search of the live pathology database to identify current clinical samples for the study.

## MATERIALS AND METHODS

Crystal Reports version 8.5 software (Seagate Software, Inc.) was used to conduct a daily search of the live pathology database of Montefiore Medical Center for patients with completed endocervical swab AC2 results who also had a SP cervicovaginal sample collected at the same visit. Montefiore Medical Center is a major tertiary hospital with an extensive network of outpatient clinics that serves an urban population with a high prevalence of sexually transmitted disease. SP samples from patients whose endocervical swab specimens tested positive for CT, GC or both, and a random selection of patients whose endocervical swab specimens tested negative for both were selected for the study.

Endocervical swab samples had been collected with the APTIMA^®^ Unisex Swab Specimen Collection Kit for Endocervical Swab Specimens (Gen-Probe Inc., San Diego, CA) and were submitted directly to virology for AC2 testing. The SP cervical specimens had been taken with a Cervex-Brush^®^ (Rovers Medical Devices B.V. Oss, The Netherlands), the head of which was detached into a specimen vial containing 10 ml of SP Preservative Fluid (TriPath Imaging Inc., Burlington, NC). In the cytology lab, the SP specimen vials were first sampled by the SP Prepmate device, which draws off 8 ml to be processed for cytology and subsequent high risk HPV testing (Hybrid Capture 2), where indicated. The remaining samples, still in the SP Preservative Fluid in the original perforated specimen vials, were kept at room temperature in a covered plastic tray. Prior to sampling, each vial was covered with parafilm and vortexed, and 1.5 ml of sample was transferred to 2.9 ml APTIMA^®^ Specimen Transfer tubes labeled only with the study sample number and without patient identifiers. Dates of specimen collection and transfer were tracked. SP samples with less than 1.5 ml remaining in the specimen vial (10 cases), together with samples more than 7 days old from date collection to transfer were excluded. The time to transfer of the SP specimens into the APTIMA transfer medium ranged from 2 to 7 days (mean 4 days, with 278 specimens or 92.7% transferred by day 5).

Once transferred into the APTIMA transfer medium, samples were refrigerated at 4°C until testing using the Tigris DTS System (Gen-probe, San Diego, CA). Samples were run in duplicate with appropriate positive and negative controls. Only after the SP results were finalized were they compared with the previously reported AC2 endocervical swab CT and GC results.

Sensitivity and specificity for the SP results were calculated taking the reported AC2 endocervical swab results as the patient’s infection status. Fisher’s exact test was used to calculate *P* values.

## RESULTS

Over a three month period, SP specimens from 300 patients aged 15–70 years (median age 23 years) were tested, 188 of whom had CT-positive and 19 of whom had GC-positive endocervical swab samples taken at the same visit (including 7 CT and GC coinfected patients). SP versus endocervical swab results are displayed in Table 1. Excluding two equivocal SP results, AC2 testing of the SP samples for CT had a sensitivity of 89.3% (CI 84.0%-93.3%) and specificity of 100.0% (CI 96.7%-100.0%). When the CT data were grouped and reanalyzed for patients up to and including 25 years old (130 swab CT positive) and patients older than 25 years (57 swab CT positive) [Table 1], the sensitivity for patients 25 years and younger was 93.1% (CI 87.2%-96.8%), which was significantly higher than the sensitivity for patients older than 25 years (80.7%, CI 68.1-90.0%, p = 0.02).

SP GC results from all 19 endocervical swab GC-positive patients were positive, and the SP GC results from all 281 GC-negative patients were negative [Table 1]. Thus, AC2 testing of SP samples for GC was 100.0% sensitive (CI 82.4%-100%) and 100.0% specific (CI 98.7%-100.0%).

**Table 1:** APTIMA AC2 Chlamydia (CT) and Gonorrhea (GC) Results - Endocervical Swab vs. SurePath (SP) Samples^a^

	*SP CT positive (n = 167)*	*SP CT negative (n = 131)*	*SP CT Sensitivity/Specificity (%)*
CT Results			
Swab CT Positive (n = 187)	167	20	Sensitivity 89.3 (95% CI 84.0-93.3)
≤ 25 yr (n = 125)	121	9	Sensitivity 93.1 (95% CI 87.2-96.8)
> 25 yr (n = 62)	46	11	Sensitivity 80.7 (95% CI 68.1-90.0)
Swab CT negative (n = 111)	0	111	Specificity 100.0 (95% CI 96.7-100.0)
GC Results			
Swab GC positive (n = 19)	19	0	Sensitivity 100.0 (95% CI 82.4-100.0)
Swab GC negative (n = 281)	0	281	Specificity 100.0 (95% CI 98.7-100.0)

a Two SP equivocal results, one endocervical swab CT positive, and one endocervical swab CT negative excluded

## DISCUSSION

Overall, AC2 testing of 1.5 ml SP samples for CT had a sensitivity of 89.3% (CI 84.0%-93.3%) and a specificity of 100% (CI 96.7%-100.0%). This sensitivity for CT is slightly lower than the 93.1% reported for AC2 on ThinPrep samples,[8] but confirms the conclusion of two earlier studies that AC2 testing of SP samples yield acceptable results.[9,10] Our sensitivity of 89.3% is slightly higher than the sensitivities of 80.0% and 85.2% reported for AC2 on SP samples sampled with the Cervex-Brush^®^ in two previous studies. The first of these prior SP studies reported rare false positive CT results, most from a single collection site among their 1615 patients, which were attributed to errors in specimen collection or processing. There were no false positives among our 111 CT swab negative cases.

Although the number of swab GC-positive cases was low (n = 19), AC2 testing of 1.5 ml SurePath samples for GC had a sensitivity of 100.0% (CI 82.4%-100.0%) and a specificity of 100.0% (CI 98.7%-100.0%). This compares favorably with the 100.0% sensitivity and specificity reported on Thin Prep[8] and 92.5% sensitivity, 100.0% specificity reported on SurePath samples tested with AC2.[9]

Several factors may have contributed to the slightly decreased sensitivity of the SP samples in testing for CT. First to consider are differences in sampling related to the different sampling devices (Cervex-Brush^®^ vs. swab). Although no study has compared these two devices specifically, a prior study comparing ACS testing of SP samples taken with the Cervex-Brush^®^ vs. a combination of the Medscand Pap Perfect^®^ plastic spatula and the Cytobrush^®^ (CooperSurgical, Trumbull, CT) found no significant difference between samples taken with these devices.[10] This suggests that the slightly higher sensitivity reported for AC2 testing of ThinPrep specimens[8] is not device-related. However, the device comparison included only 25 CT-positive patients, which may be an insufficient number to detect a small device-related difference. Sample order, not known in the current study, is another possible factor, although one prior study of the effect of endocervical swab collection order on samples tested for CT by DNA probe (PACE 2), ligase chain reaction, and PCR found that the order of swab collection from the endocervix did not influence the test results.[11] Another possible factor is that the SP sample is initially diluted in 10ml of SP fixative, as opposed to 2.9 ml of fixative in the APTIMA collection vial. In patients with very small numbers of CT, this could possibly lead to false negative results due to uneven distribution of the cellular material. A related factor is the volume of the SP specimen aliquoted for AC2 testing. The volume remaining in the SurePath vial after sampling by the SurePath Prepmate can be up to 2ml but varies. We tested 1.5ml because most patient vials had at least this volume remaining (10 samples or approximately 3% were rejected for lesser volumes, ranging from 0.9-1.2ml). Use of a greater volume wherever possible may increase sensitivity, as has been demonstrated on Thinprep samples tested for CT and GC using the COBAS AMPLICOR PCR system (Roche Diagnostic Systems, Pleasanton, CA).[4] On the other hand, a prior study showing that mocked SP samples were positive for CT to a dilution of 10^-9^ suggests that sample volume and dilution may not be critical factors.[10]

Finally, the sensitivity of the test may also be influenced by the delay in transferring the SurePath sample into the APTIMA transfer tube. The APTIMA transfer medium is designed to release RNA from the cells and stabilize it until testing. The effect on RNA of the SP preservative, which contains <1% of formalin in addition to <24% denatured ethanol and 1.2% methanol, is not fully known. A recent study by Chernesky *et al*, that included positive pooled patient SP specimens tested by AC2 showed that all of the 5 pooled specimens remained positive for CT for 10 days at room temperature.[10] As Chernesky *et al*., note, their results differed from a previous study of mocked specimens stored in SP preservative at room temperature and tested over a three week period for HPV RNA using Trizol or Quiagen extraction and quantitative PCR. That earlier study reported a severe drop in HPV RNA recovery, most of which occurred in the first six days, which they attributed to RNA degradation.[12] However, such a severe drop is at odds with the high AC2 CT sensitivity observed in this and prior studies.[9,10] As Chernesky *et al*. observed, the much improved RNA stability noted in their study may be due to the transfer of SP samples into the APTIMA transfer medium, “providing stability to the amplification target” prior to extraction. Nevertheless, two of the samples in their study showed a gradual decline in RLU to >60% of the initial value by day ten,[10] suggesting that some degradation may be occurring. A similar decline might lead to false negative results in patients with low numbers of organisms.

While the exact reason for slightly lower sensitivity is unclear, our data do suggest that patient age may influence the results. Among our patients, the sensitivity was higher in patients 25 years and younger (sensitivity 93.1%, CI 87.2%-96.8%) than in patients older than 25 (80.7%, CI 68.1%-90.0%). The decreased sensitivity in patients over 25 years may relate to less active infection and lower numbers of organisms in the older age group and the difficulty of obtaining adequate material from a chronically infected cervix due to fibrosis and stenosis. However, although these results seem biologically plausible, they are based on a small sample size in the >25 year age group. A larger study is needed to determine if these findings are generalizable.

Finally this study illustrates the potential of search in a live LIS database to identify current patient samples for a prospective study. The ability to search for patients with concurrent AC2 and SurePath samples and to target known CT and GC positive, and random negative samples made it possible to conduct this study without enrolling patients in a large multi-center study. With regard to potential bias introduced by the analysis of samples with previously known endocervical swab results, if any bias is introduced it would be a negative one. Unlike a prospective study, in which special care would be taken in obtaining and transporting patient samples, in this study the clinicians who obtained the samples were unaware that they would be part of a comparison study. If anything, this might introduce a negative bias, where the sensitivity of the test might be reduced due to sampling error. Such use of the live database to select samples based on known results and other parameters will have a significant impact on research done in the future, and, when an LIS database can be used in a similar fashion, cost savings can be significant.

## CONCLUSIONS

In summary, AC2 testing of SP samples transferred to the APTIMA transfer medium within seven days of collection is highly sensitive and specific for detecting GC infection. It also is highly sensitive and specific for detecting CT in patients under 25 years, which is precisely the age group that is at highest risk for CT infection and its multiple associated comorbidities including PID, ectopic pregnancies and infertility. In older patients AC2 testing of SurePath samples may be somewhat less sensitive but, recognizing that false negative results may occur, it still may be utilized for screening purposes and in cases where endocervical swab testing was overlooked. Use of a larger volume of the sample remaining in the SurePath specimen vial and minimizing the time to transfer SP sample may further enhance sensitivity of the test. Conversely, testing of smaller volumes (3% of specimens in our lab) or of samples stored in the SP fixative at room temperature for periods longer than seven days prior may yield lower sensitivities than reported in this study.

## COMPETING INTEREST STATEMENT BY ALL AUTHORS

No competing interest to declare by any of the authors.

## AUTHORSHIP STATEMENT BY ALL AUTHORS

Each author acknowledges that this final version was read and approved. All authors of this article declare that we qualify for authorship as defined by ICMJE http://www.icmje.org/#author. Each author has participated sufficiently in the work and take public responsibility for appropriate portions of the content of this article.

## ETHICS STATEMENT BY ALL AUTHORS

This study was conducted with approval from Institutional Review Board (IRB) of the institution associated with this study. Authors take responsibility to maintain relevant documentation in this respect.

## EDITORIAL / PEER-REVIEW STATEMENT

To ensure integrity and highest quality of CytoJournal publications, the review process of this manuscript was conducted under a double blind model (authors are blinded for reviewers and reviewers are blinded for authors) through automatic online system.
